# Epidemiological and genomic features of chikungunya virus disease in travellers returning from Cuba, September 2025 to January 2026: a GeoSentinel analysis

**DOI:** 10.2807/1560-7917.ES.2026.31.16.2600286

**Published:** 2026-04-23

**Authors:** Marta Díaz-Menéndez, Concetta Castilletti, Kevin O'Laughlin, Oula Itani, Hilmir Asgeirsson, Ana Vázquez González, Francesca F. Norman, Cecilia Perret, Camilla Rothe, Michela Deiana, Simone Malagò, Victor Max Corman, Gabriela Equihua Martinez, Sami Alcedo, Eva Clark, Silvia Odolini, Daniel Camprubí Ferrer, Federico Giovanni Gobbi, Davidson H. Hamer, Carsten Schade Larsen, Claudia Beisel, Obinna Nnedu, Diana Pou Ciruelo, Graciela Rodriguez Sevilla, Fernando de la Calle-Prieto, Hannah Emetulu, Michael Libman, Milan Trojánek, Stephen D. Vaughan, Ralph Huits

**Affiliations:** 1 National Referral Centre for Tropical Diseases and International Health, IdiPaz, Hospital Universitario La Paz-Carlos III Madrid, CIBERINFEC, Madrid, Spain; 2 Department of Infectious, Tropical Diseases and Microbiology, IRCCS Sacro Cuore Don Calabria Hospital, Negrar di Valpolicella, Italy; 3 Division of Global Migration and Health, Centers for Disease Control and Prevention, Atlanta, Georgia, United States; 4 Institut Pasteur, Centre Médical, Centre d'Infectiologie Necker-Pasteur, Paris, France; 5 Department of Infectious Diseases, Karolinska University Hospital, Stockholm, Sweden; 6 Center for Infectious Medicine, Department of Medicine Huddinge, Karolinska Institutet, Stockholm, Sweden; 7 Arboviruses and Viral Imported Diseases Laboratory, Centro Nacional de Microbiologia, Instituto de Salud Carlos III, Majadahonda, Madrid, Spain; 8 Centro de Investigación Biomédica en Red de Epidemiología y Salud Pública (CIBERESP), Madrid, Spain; 9 National Referral Unit for Tropical Diseases. Infectious Diseases Department. Ramón y Cajal University Hospital, IRYCIS, CIBERINFEC, Madrid, Spain; 10 School of Medicine, Pontificia Universidad Católica de Chile. SENTINET: Surveillance, Epidemiology, and New Technologies for Infectious Emerging Threats, Santiago, Chile; 11 Institute for Infectious Diseases and Tropical Medicine, LMU University Hospital, Munich, Germany; 12 PhD National Programme in One Health Approaches to Infectious Diseases and Life Science Research, Department of Public Health, Experimental and Forensic Medicine, University of Pavia, Pavia, Italy; 13 Institute of International Health, Charité – Universitätsmedizin Berlin, corporate member of Freie Universität Berlin and Humboldt Universität zu Berlin, Charité Center for Global Health, Berlin, Germany; 14 Department of Clinical Sciences, Institute of Tropical Medicine, Antwerp, Belgium; 15 Departments of Medicine (Infectious Diseases) and Pediatrics (Tropical Medicine), National School of Tropical Medicine, Baylor College of Medicine, Texas, Houston, United States; 16 University Division of Infectious and Tropical Diseases, ASST Spedali Civili Hospital, Brescia, Italy; 17 Barcelona Institute for Global Health (ISGlobal), Barcelona, Spain; 18 International Health Department, Hospital Clínic de Barcelona, Barcelona, Spain; 19 Facultat de Medicina i Ciències de la Salut, Universitat de Barcelona (UB), Barcelona, Spain; 20 Department of Clinical and Experimental Sciences, University of Brescia, Brescia, Italy; 21 Department of Global Health, Boston University School of Public Health; Section of Infectious Diseases, Department of Medicine, Boston University Chobanian & Avedisian School of Medicine; Center on Emerging Infectious Disease and National Emerging Infectious Diseases Laboratory, Boston University, Massachusetts, Boston, United States; 22 Department of Clinical Medicin, Aarhus University; Department of Infectious Diseases, Aarhus University Hospital, Aarhus, Denmark; 23 Division of Infectious Disease and Tropical Medicine, Heidelberg University Hospital, Heidelberg, Germany and German Center for Infection Research (DZIF), partner site Heidelberg University Hospital, Heidelberg, Germany; 24 Department of Infectious Diseases. Ochsner Medical Center, Louisiana, New Orleans, United States; 25 Drassanes-Vall d'Hebron Center for International Health and Infectious Diseases PROSICS Barcelona, Department of Infectious Diseases, Hospital Universitari Vall d'Hebron, Universitat Autònoma de Barcelona, Barcelona, Spain; 26 Hospital Universitario La Paz, Madrid, Spain.; 27 GeoSentinel, Georgia, Atlanta, United States; 28 J.D. MacLean Centre for Tropical and Geographic Medicine, McGill University, Montreal, Canada; 29 Department of Infectious Diseases and Travel Medicine, Second Faculty of Medicine, Charles University and University Hospital Motol, Prague, Czechia; 30 Department of Medicine, Division of Infectious Diseases, University of Calgary, Alberta, Calgary, Canada

**Keywords:** Chikungunya virus, GeoSentinel Surveillance, Aedes, Phylogenetic analysis, Travel-associated infections

## Abstract

During September 2025–January 2026, 111 travellers (61 female/50 male; median age:  53 years) who acquired chikungunya virus (CHIKV) in Cuba were reported to GeoSentinel. Upon return, 64.2% (70/109) were potentially viraemic. Only 8.2% (9/98) had received pre-travel consultations. The CHIKV was of East-Central-South Africa genotype, closely related to Brazilian strains. International travellers can serve as arboviral outbreak sentinels and, if viraemic, risk introducing CHIKV into areas with established *Aedes* spp. vectors. Their effective surveillance can trigger adequate public health responses.

GeoSentinel is a global clinician-based sentinel surveillance network of specialised travel and tropical medicine sites across six continents (https://geosentinel.org/sites/). From September 2025, sites in this network identified an increasing number of travellers returning from Cuba with chikungunya virus (CHIKV) infection, consistent with a large outbreak there, which was later reported by the Pan American Health Organization [1,2]. Here, we describe the cases of travel-associated chikungunya acquired in Cuba, which were reported to GeoSentinel during the outbreak period. We also phylogenetically analyse CHIKV genome sequences derived from viraemic travellers.

## Characteristics of travellers 

**Figure 1:**
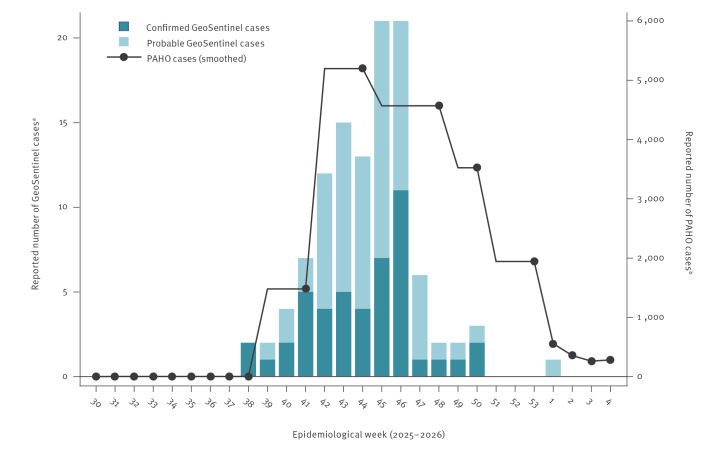
Number of chikungunya cases reported to GeoSentinel (n = 111) and the Pan American Health Organization (n = 163,625), by epidemiologic week, Cuba, 2025–2026

Between September 2025 and January 2026, 111 travellers from 13 countries with chikungunya acquired in Cuba were reported to the GeoSentinel Surveillance Network (Figure 1). Confirmed cases were positive by a CHIKV-specific nucleic acid amplification test, comprising either PCR, reverse transcription-PCR (RT-PCR) or loop-mediated isothermal amplification, and/or had documented anti-CHIKV immunoglobulin (Ig)G seroconversion. Probable cases were defined by a single positive CHIKV antibody finding, including an IgM and/or IgG positive result, with compatible clinical presentation and exposure [3]. Potentially viraemic travellers were defined in two ways, including having an RT-PCR positive result when tested after return from Cuba, or having illness onset occurring within 8 days before the departure from Cuba or on/after the departure date.

PAHO: Pan American Health Organization.

^a^ GeoSentinel chikungunya cases (n = 111) are plotted by week of illness onset when available; otherwise, the week of initial clinical visit was used.

^b^ Surveillance data from PAHO for chikungunya infections detected in Cuba are reported at irregular intervals (2025 week 30: 8 cases; week 38: 34 cases; week 41: 4,472 cases; week 44: 20,062 cases; week 48: 38,342 cases; week 50: 45,388 cases; week 53: 51,217 cases. 2026 week 1: 557 cases; week 2: 913 cases; week 3: 1,175 cases; week 4: 1,457 cases). The numbers of reported cases moreover include both confirmed and probable cases. For visualisation purposes, if numbers of cases are provided in a week following one or more weeks without reported cases, the number of cases in that reporting week are distributed evenly across the preceding weeks lacking data, assuming constant weekly incidence in those weeks and the reporting week.

Case counts before week 30 2025 are not included.

The travellers’ median age was 53 years (range: 5–78); 55.0% were female (with the rest being male); 49.6% were tourists and 34.3% were visiting friends and relatives (Table). The median length of stay in Cuba was 20.5 days (interquartile range (IQR): 13.0–31.0). All travellers reported symptoms compatible with chikungunya and most (83.5%) fell ill during their stay in Cuba or return journey; 96.4% were managed as outpatients. The median interval between symptom onset and evaluation at a GeoSentinel site was 16 days (IQR: 6.0–39.0). Overall, 70 of 109 travellers with reliable information (64.2%) were considered potentially viraemic at the time of return travel. Among GeoSentinel countries outside of Cuba detecting cases, Spain reported most (n = 50), followed by Germany (n = 29), while the remainder each reported seven cases or less. Only eight of 98 travellers with available data (8.2%) had received a pre-travel consultation.

**Table:** Epidemiological, travel and clinical characteristics of travellers with chikungunya virus infection imported from Cuba, GeoSentinel Surveillance Network, September 2025–January 2026 (n = 111)

Characteristic	Values		
**Demographics**			
Median age in years (range)	53 (5–78)		
Female sex, number and %	61	55.0	
Male sex, number and %	50	45.0	
**Reporting countries, number of cases**			
Spain	51		
Germany	29		
Italy	7		
Belgium	6		
Chile, France, United States	3 (each)		
Canada, Czechia, the Netherlands	2 (each)		
Denmark, Sweden, Switzerland	1 (each)		
**Reason for travel, number and %**			
Tourism	55	49.6	
Visiting friends and relatives	38	34.3	
Business^a^	7	6.3	
Cuban residents diagnosed during travel abroad	5	4.5	
Other/not ascertainable	6	5.4	
**Diagnosis^b^**, **number and %**			
Confirmed	46	41.4	
Probable	65	58.6	
**Reported symptoms, number and %**			
At least one symptom	111		100
Rheumatic and musculoskeletal (including arthralgia, myalgia, arthritis)	107		96.4
Constitutional symptoms (fever/sweats/chills)	92		82.9
Dermatological (including diffuse or focal rash, itch, lesion)	53		47.8
Other^c^	70		63.1
**Setting, number and %**			
Outpatient	107		96.4
Inpatient	4		3.6
**Pre-travel consultation, number and %**			
No	90		81.1
Yes	8		7.2
Unknown/not applicable	13		11.7
**Length of stay in Cuba^d^, median days (IQR)**	20.5 (13.0–31.0)		
Tourism	15.0 (13.0–23.0)		
Visiting friends and relatives	26.5 (16.0–49.0)		
Business	85.0 (6.0–181.0)		
Other/not ascertainable	26.0 (10.0–61.0)		
**Timing of disease onset, number and %^e^**			
During stay in Cuba or return trip	91		83.5
After trip to Cuba	18		16.5
**Potential viraemic importation**, **number and %**	70		64.2^e^
Illness onset ≤ 8 days before departure	41		58.6^f^
Illness onset after the day of departure	18		25.7^f^
Illness onset on the day of departure	11		15.7^f^
RT-PCR positive	36^g^		51.4^f^
**Potential viraemia by travel type, number and % within travel type** ^h^			
Tourism	35		64.8^h^
Visiting friends and relatives	23		60.5^h^
Cuban residents diagnosed during travel abroad	4		80.0^h^
Business	4		66.7^h^
Other/not ascertainable	4		66.7^h^
**Time elapsed between symptom onset and evaluation at a GeoSentinel site**			
Median days (IQR)	16 (6.0–39.0)		

Ig: immunoglobulin; IQR: interquartile range; LAMP: loop-mediated isothermal amplification; NAAT: nucleic acid amplification test; RT-PCR: reverse transcription PCR.

^a^ Business travel was professional, corporate or included travel to a conference.

^b^ Confirmed included positive NAAT, including PCR, LAMP, RT-PCR (n = 42) and paired serology (n = 4). Probable included positive single serology (IgM and IgG) (n = 47), single serology (IgM only) (n = 14), single serology (IgG only) (n = 1), and single antibody results (unspecified).

^c^ Other symptoms included abdominal pain/discomfort, acute diarrhoea, anorexia, cardiac symptoms, conjunctivitis, cough, dizziness, dysuria, eye symptoms, headache, loss of consciousness/syncope, lymphadenopathy, nausea, neck stiffness/photophobia, psychological symptoms, runny nose/sore throat, vomiting, and other.

^d^ This analysis included 104 cases, because Cuban residents diagnosed abroad were excluded (n = 5), as well as a case with missing illness onset date (n = 1) and another with data entry error on illness onset date (n = 1). Included cases were tourists (n = 54) and people visiting friends and relatives (n = 38), travelling for business (n = 6) or for other/not ascertainable reasons (n = 6).

^e^ Travellers were considered potentially viraemic if illness onset date was within 8 days of crossing a border. The denominator for the percentages (n = 109) did not account for two cases, which were either excluded because the illness onset date was missing (n = 1) or had a data entry error (n = 1). Cuban residents diagnosed during travel abroad were included if illness onset date minus arrival date ≤ 8 days.

^f^ Percentages are based on the denominator of 70 travellers considered potentially viraemic at the time of border crossing.

^g^ The number includes only those that were RT-PCR positive among the 70 travellers considered potentially viraemic at the time of border crossing.

^h^ The denominators of the percentages can be found in the ‘Reason for travel’ section of the table.

### Phylogenetic analysis 

**Figure 2:**
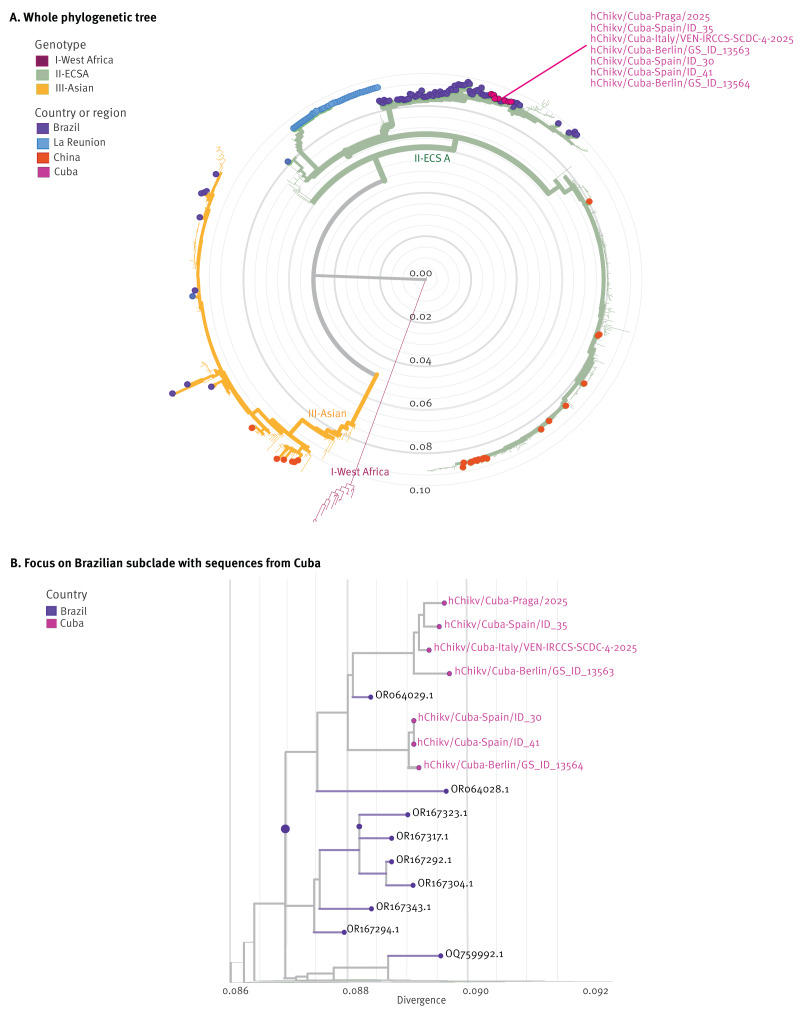
Phylogenetic tree based on complete chikungunya virus genome sequences, showing those from travellers from Cuba (A) to be of the ECSA-II lineage and (B) segregating within a Brazilian subclade, 2025 (n = 7 travellers’ sequences analysed)

We obtained genome sequences of seven CHIKV strains derived from cases who had travelled to Cuba. Sequencing was performed at laboratories in Germany, Spain, Italy, and Canada. Phylogenetic analysis, comparing consensus sequences with CHIKV sequences publicly available in the National Center for Biotechnology Information (NCBI) and GISAID repositories (https://www.eurosurveillance.org/www.gisaid.org), showed that strains circulating in Cuba in 2025 belonged to the East-Central-South African (ECSA) genotype, and clustered in ECSA-II lineage (Figure 2), where they were most closely related to strains detected during the 2023–2024 outbreak in São Paulo State, Brazil [4,5].

ECSA: East-Central-South Africa.

A. Comprehensive tree showing the three major CHIKV outbreaks reported during 2024–2025 (Réunion Island, Brazil and China) including strains belonging to ECSA and Asian genotypes. The tree is rooted using West African strains.

B. Zoomed view of the Brazilian subclade highlighting seven imported cases from Cuba.

Light-grey circles in panel A, and vertical scale bars in panel B indicate genetic divergence between nucleotides sequences. Branches are coloured according to genotype, and tips (i.e. leaves) are coloured according to the country of origin of the sequences. Multiple sequence alignment and phylogenetic construction were performed using *Nextclade* (https://clades.nextstrain.org) and trees were visualised using *Auspice* (auspice.us 0.13.0).

## Discussion

Following the first autochthonous chikungunya cases reported in Cuba during the 2014 Caribbean epidemic [6], CHIKV circulation in the country remained limited, with only small, localised re-emergence observed in 2023–2024 [7]. The detection of 111 CHIKV infections in international travellers from Cuba within a 4-month period contrasts sharply with preceding years, when there were only 10 Cuba travel-associated chikungunya cases reported to GeoSentinel since its introduction in the country [8]. This supports the occurrence of intense transmission during the 2025–2026 outbreak [1]. Travellers can act as sentinels of outbreaks in settings where local or regional surveillance data may be delayed or incomplete, particularly for arboviral diseases, for which laboratory differentiation can be limited and cases may be reported under broader syndromic categories such as suspected dengue [9].

From a clinical and public health perspective, this outbreak is noteworthy given relatively limited chikungunya activity in the Caribbean since the 2014–2015 epidemics [6]. The re-emergence of high case numbers in Cuba aligns with patterns observed in other large outbreaks, where waning population immunity and changes in vector density or competence have been implicated, as well as broader contextual conditions affecting outbreak detection and response capacity, such as surveillance sensitivity, the timeliness of control measures and environmental conditions influencing transmission [10,11]. Notably, the numbers of reported chikungunya-associated deaths (46 deaths by 31 December 2025) are in keeping with levels described in previous outbreaks [1].

These findings underscore the importance of accurate diagnostics in settings such as Cuba, where multiple arboviruses, including dengue and Oropouche viruses, co-circulate [12]. The median interval between symptom onset and evaluation at a GeoSentinel site was 16 days, suggesting that some travellers may have sought care after the acute phase, possibly because of persistent symptoms and/or delayed arboviral differentiation in routine practice. Overlapping clinical presentations may complicate syndromic diagnosis, affecting patient management, surveillance accuracy, and interpretation of outbreak dynamics [13]. Timely access to specific diagnostic testing is therefore essential to distinguish co-circulating arboviral infections and to accurately characterise the scale and impact of each outbreak. GeoSentinel may assist in filling these gaps by detecting travel-related infections and reporting these as sentinel events. In the current event, signals of a possible outbreak in Cuba were shared within the GeoSentinel network in September 2025 before the full scale of the outbreak had been formally characterised.

Phylogenetic analysis of viral genomes obtained during outbreaks complements traditional surveillance by elucidating spatial and temporal transmission patterns [14]. We identified an ECSA-II lineage CHIKV in Cuba, with strains closely related to those circulating in São Paulo State, Brazil, during a 2023–2024 outbreak there. This finding is compatible with broader regional viral movement, although the precise route of introduction into Cuba cannot be inferred from the available genomic data, particularly given limited virological surveillance in parts of Latin America [15]. Similarly, genomic characterisation of Oropouche virus from a traveller returning from Cuba to Italy showed clustering with strains linked to the 2022–2024 Oropouche Brazilian outbreak, suggesting that regional viral lineages may reach Cuba through under-recognised transmission pathways [16]. Surveillance of infected travellers can therefore contribute to detecting viral introductions and understanding local transmission dynamics in settings with limited molecular surveillance capacity [17].

This outbreak’s implications extend beyond the country of exposure, particularly for Spain, which reported the highest number of cases in this series and, at the same time has widespread well-established populations of *Aedes albopictus* which can serve as CHIKV vector [18]. Although no autochthonous CHIKV transmission has been documented in Spain to date [19], recent locally acquired dengue cases indicate that ecological conditions for arboviral transmission are already present [20]. Importation of viraemic travellers, regardless of travel reason, during an outbreak period may therefore represent a plausible risk scenario for CHIKV introduction. Notably, our analysis suggests that more than half of the patients described in this report were likely to be viraemic after departure from Cuba. This, moreover, does not take into account asymptomatic viraemic patients, as GeoSentinel captures only symptomatic travellers seeking care [21]. The absence of documented autochthonous transmission in Spain despite the large number of travel-associated cases may partly reflect the timing of the outbreak, as most importations occurred in late autumn and winter, outside the main season of *Ae. albopictus* activity in much of Spain.

Experience from other European countries supports the concern of introduction risk. In 2025 in mainland Europe, 1,172 autochthonous chikungunya cases were reported, exceeding the total number in the previous 17 years [19,22]. Increased chikungunya importations from the Indian Ocean region, including Réunion, have highlighted how traveller-associated cases can serve as early indicators of transmission in the country of exposure and signal potential risks for areas with competent vectors in the country of further travel or return [23]. The occurrence of local CHIKV transmission in parts of the United States from 2014 onwards, after only travel-associated cases had been observed, further illustrates the potential for autochthonous spread following virus introduction into areas with competent vectors [24]. In this context, timely recognition of imported infections, targeted public health notification, and post-travel counselling on mosquito avoidance should be considered key components of secondary prevention strategies in non-endemic countries where relevant *Aedes* spp. are present, particularly during periods of vector activity.

Several limitations of the current should be acknowledged. GeoSentinel surveillance is not population-based and does not allow estimation of attack rates or comparison with travel denominators. Clinical and laboratory data are collected as part of routine practice and may be incomplete, particularly regarding long-term outcomes. In addition, systematic testing for other co-circulating arboviruses was not performed in all patients, precluding reliable assessment of co-infections. Nevertheless, consistent detection of unusual case numbers across multiple countries over a short period suggests that these imported cases may represent a true epidemiological signal rather than a reporting artefact.

Knowledge of potential importations is important, as beyond acute presentation, CHIKV infection is associated with prolonged and sometimes disabling musculoskeletal sequelae, which may persist for months or years [25]. This longer-term burden may extend beyond peak outbreak timeframes and place additional demands on healthcare systems in non-endemic countries. Clinicians should therefore remain alert, not only to acute chikungunya in returning travellers, but also to the potential of further chronic manifestations, ensuring appropriate follow-up and management. Strengthening awareness and recognition of post-chikungunya syndromes will be essential to mitigate its longer-term clinical impacts.

In terms of prevention, the recent licensing of two chikungunya vaccines in a number of countries also provides an opportunity for intervention and evaluation of their risks and benefits in high transmission scenario [26]. Pretravel consultation, sought by a small minority of travellers in this series (8.2%), also represents a key occasion to advise on outbreak risks, vaccination eligibility, and mosquito bite avoidance both during travel and after return if febrile or otherwise symptomatic, particularly in areas with competent *Aedes* vectors. Pre-travel advice should also include healthcare-seeking recommendations and temporary deferral of blood donation after travel [27].

## Conclusion

The 111 travel-associated chikungunya cases acquired in Cuba between September 2025 and January 2026, which were reported to GeoSentinel, underscore the role of international travellers as sentinels of arboviral outbreaks, particularly when these occur in areas with limited epidemiological or laboratory surveillance. The event reported here also evokes the potential risk of CHIKV introduction into areas with established population of *Aedes* spp. vectors. Strengthening awareness and preparedness to recognise and address imported and autochthonous cases is essential to mitigate the spread and impact of CHIKV.

## Data Availability

Aggregate epidemiological and clinical data supporting the findings of this study are included in the article. De-identified individual-level data are not publicly available because they were collected through routine public health surveillance and are subject to data protection and institutional restrictions. CHIKV genome sequences generated in this study have been deposited in GISAID [EPI_ISL_20358255, EPI_ISL_20359055].
